# Complete heart block ensuing from a metastatic small cell carcinoma: a case report

**DOI:** 10.1186/s13256-021-03244-z

**Published:** 2022-02-10

**Authors:** Pedro Pallangyo, Garvin Kweka, Frederick Lyimo, Henry Mayala, Happiness J. Swai, Zabella Mkojera, Nsajigwa Misidai, Makrina Komba, Jalack Millinga, Smita Bhalia, Faustina Mwapinga, Salma Wibonela, Mohamed Janabi

**Affiliations:** 1 Department of Research & Training, Jakaya Kikwete Cardiac Institute, P.O Box 65141, Dar es Salaam, Tanzania; 2 Department of Internal Medicine, Muhimbili National Hospital, P.O Box 65000, Dar es Salaam, Tanzania; 3 Department of Radiology, Muhimbili National Hospital, P.O Box 65000, Dar es Salaam, Tanzania; 4 Department of Nursing, Jakaya Kikwete Cardiac Institute, P.O Box 65141, Dar es Salaam, Tanzania; 5 Department of Adult Cardiology, Jakaya Kikwete Cardiac Institute, P.O Box 65141, Dar es Salaam, Tanzania

**Keywords:** Small cell carcinoma, Oat cell carcinoma, Lung cancer, Complete heart block, Complete atrioventricular block, Third-degree heart block, Cardiac metastases, Case report

## Abstract

**Introduction:**

Notwithstanding the diagnostic and therapeutic advancements, the incidence of cardiac metastases has increased in recent decades. Lung cancers are the most common primary malignant neoplasms with cardiac metastasis potential. The clinical presentation of cardiac metastases is either silent or vague, and largely depends on the infiltrated location and tumor burden. Although arrhythmias are not uncommon in metastatic cardiac tumors, complete heart block is relatively a rare manifestation. We present a case of complete heart block due to a metastatic small cell carcinoma in a 67-year-old male of African origin.

**Case presentation:**

A 67-year-old male of African origin from rural Tanzania was referred to us for expert management. He is a retired agromechanic with over 30 years exposure to asbestos-containing brake linings. His past medical history was unremarkable, but the family-social history was evident for a heavy alcohol intake and chronic cigarette smoking. He presented with a 24-week history of progressive shortness of breath and an 8-week history of recurrent syncopal attacks coupled with a significant weight loss. He had normal echocardiographic findings, however, the electrocardiogram showed features of complete heart block. Chest X-ray showed a homogeneous opacification on the right side and computed tomography scan revealed a solid right lung mass with metastases to the liver, heart, bowels, and bone. He underwent bronchoscopy, which revealed an endobronchial mass obstructing the bronchus intermedius. Histological examination of a section of lung biopsy taken during bronchoscopy confirmed the diagnosis of a small cell carcinoma. The patient underwent dual chamber pacemaker implantation with successful sinus rhythm restoration. He made an informed refusal of chemotherapy and inevitably died 18 months post pacing.

**Conclusions:**

Despite the advancements in medical diagnostics and management, lung cancers are often diagnosed in advanced stages, with an inevitable grave prognosis. Small cell carcinoma has the potential to metastasize to the heart, resulting in complete heart block.

## Introduction

Cardiac metastases rarely complicate the course of neoplasms. Primary tumors of the heart are very rare (∼0.02%) but metastasis from various malignant neoplasms is about 40 times more common [1–3]. Diversity in the clinical presentation of cardiac malignancies is often elusive, resulting in diagnostic dilemmas, delayed diagnosis, or even misdiagnosis. Owing to the unclear, nonspecific,and largely asymptomatic presentation of cardiac metastases, antemortem diagnosis is uncommon [4].

Lung cancers are the most common (∼39%) primary malignant neoplasms with cardiac metastasis potential [2, 3, 5, 6]. While only about 10% of living lung cancer patients present with cardiac involvement, a detection rate of up to 35% is reported among autopsy cases [7, 8]. Dissemination of lung malignancies to the heart may occur in a number of ways including hematogenous spread, direct invasion, transvenous extension, and retrograde lymphatic spread [3, 9]. Although arrhythmias are not uncommon in metastatic cardiac tumors, complete heart block is a relatively infrequent manifestation [4]. We present a case of complete heart block (CHB) due to a metastatic small cell carcinoma in a 67-year-old male of African origin.

## Case presentation

A 67-year-old male of African origin from the Kilimanjaro region north of Tanzania was referred to us for expert management. He is a retired agromechanic with over 30 years exposure to asbestos-containing brake linings. His past medical history was unremarkable, but the family-social history was evident for a heavy alcohol intake (5 units/day) and chronic cigarette smoking (20 pack years). He presented with a 24-week history of progressive shortness of breath and an 8-week history of recurrent syncopal attacks. Such complaints were associated with awareness of heart beat, easy fatigability, light headedness, dry cough, and weight loss (∼15 kg in 6 months). There was a negative history of fever, night sweats, chest pain, visual disturbances, recurrent headaches, bone pain, vomiting, or diarrhea.

**Fig. 1:**
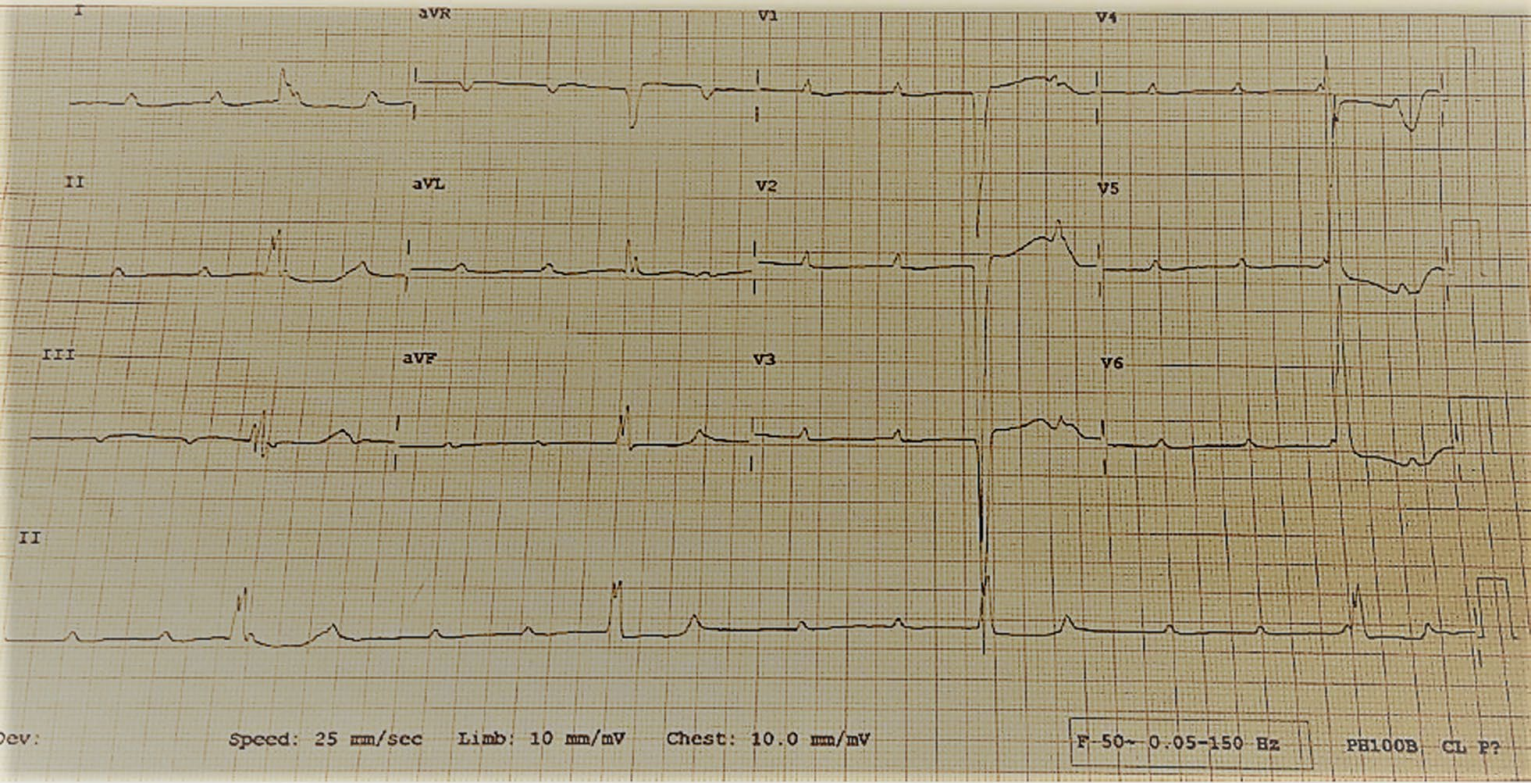
Electrocardiogram showing AV dissociation in keeping with diagnosis of complete heart block

**Fig. 2:**
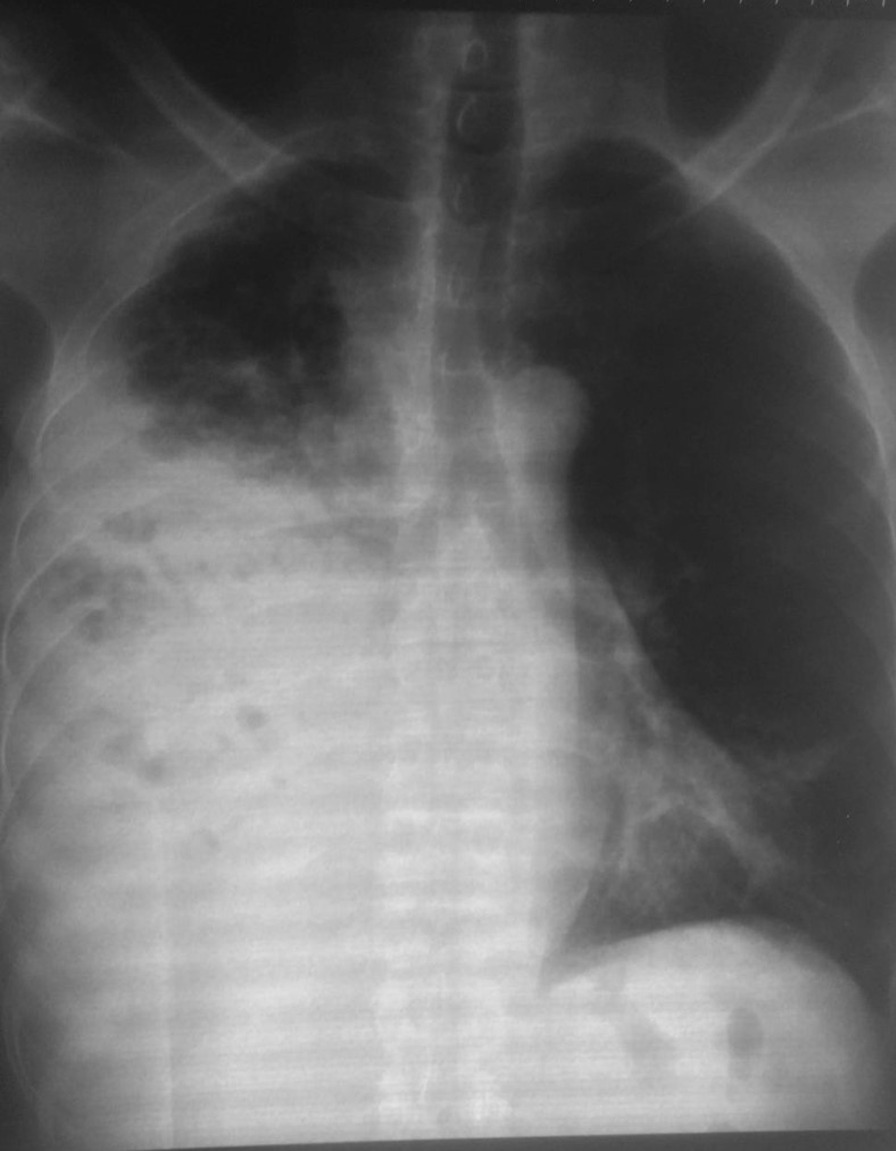
Chest x-ray (PA view) displaying consolidation on right middle to lower lung zones with areas showing bronchograms

**Fig. 3:**
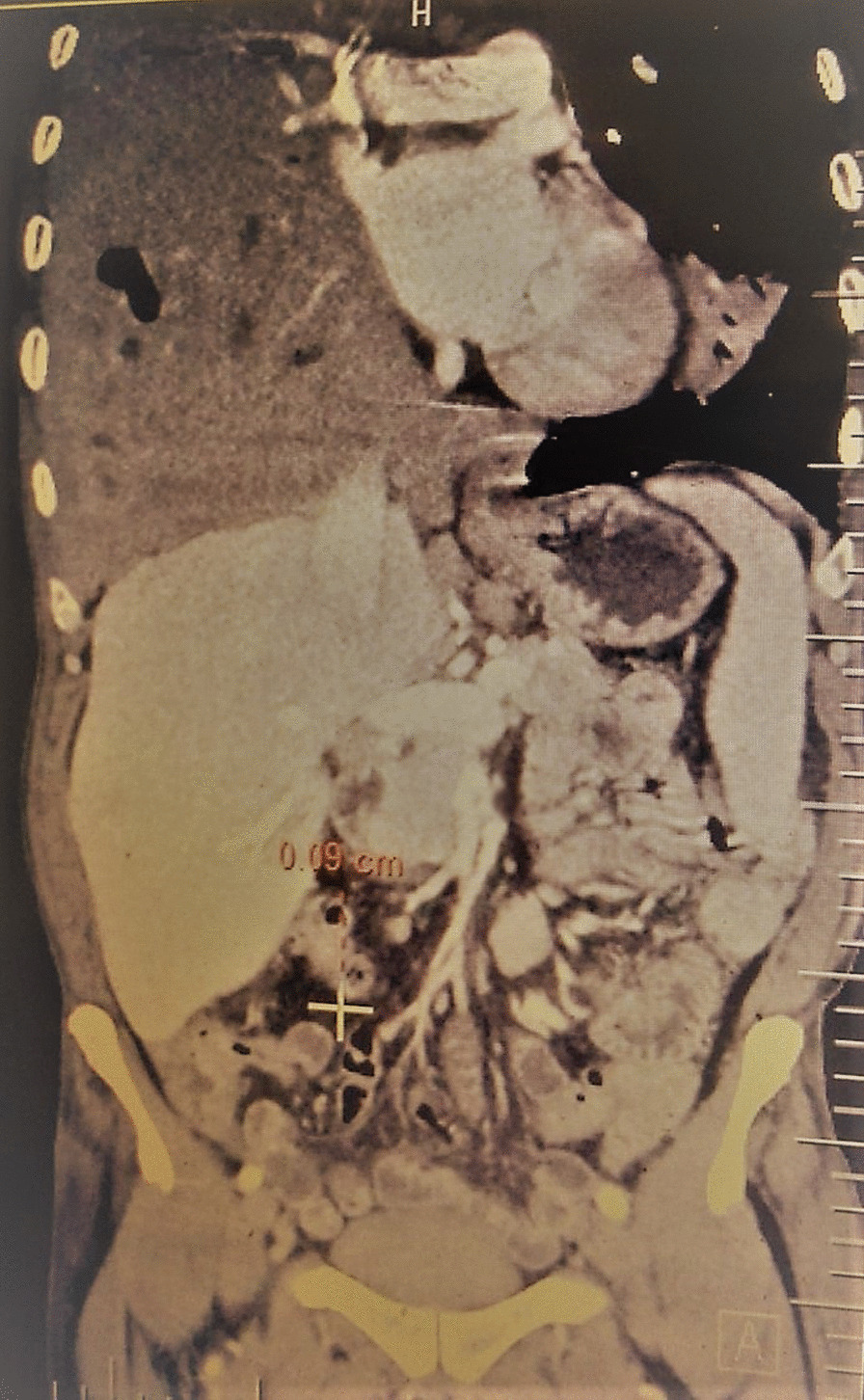
Computed tomography chest and abdomen (coronal reformatted view) displaying a mass-like dense consolidation of the right lung and an ill-defined heterogeneously hypodense lesion at the caudate lobe of the liver suggestive of liver metastasis

**Fig. 4:**
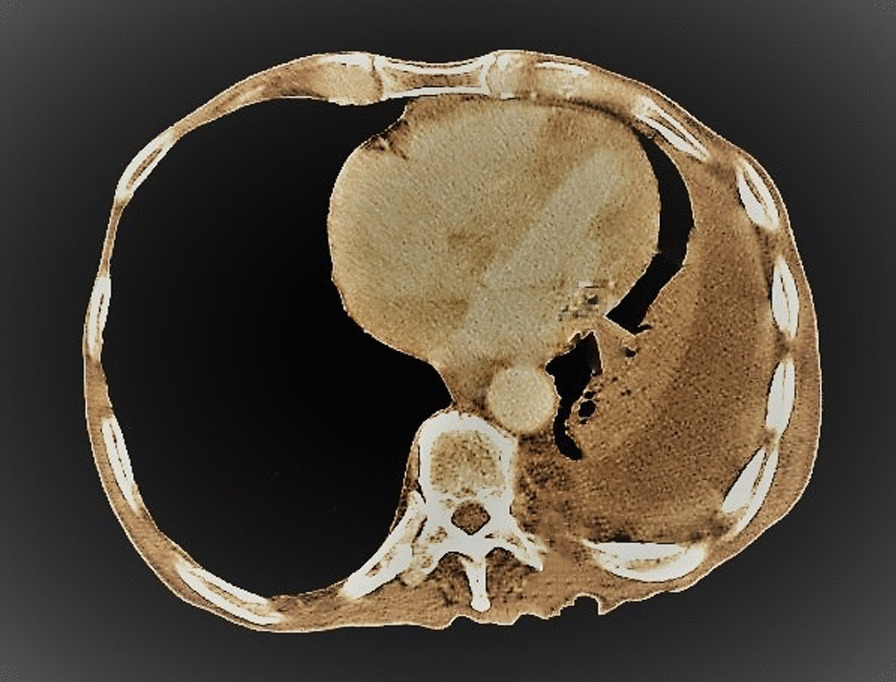
Computed tomography scan showing an intracardiac mass

**Fig. 5:**
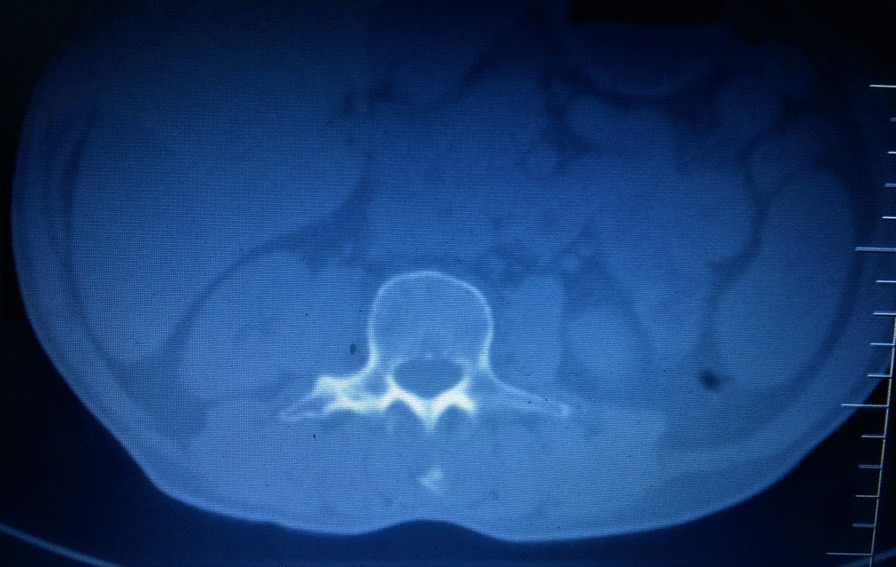
Computed tomography scan at the level of kidney displaying hyperdensity at the left pedicle suggestive of bone metastasis

**Fig. 6:**
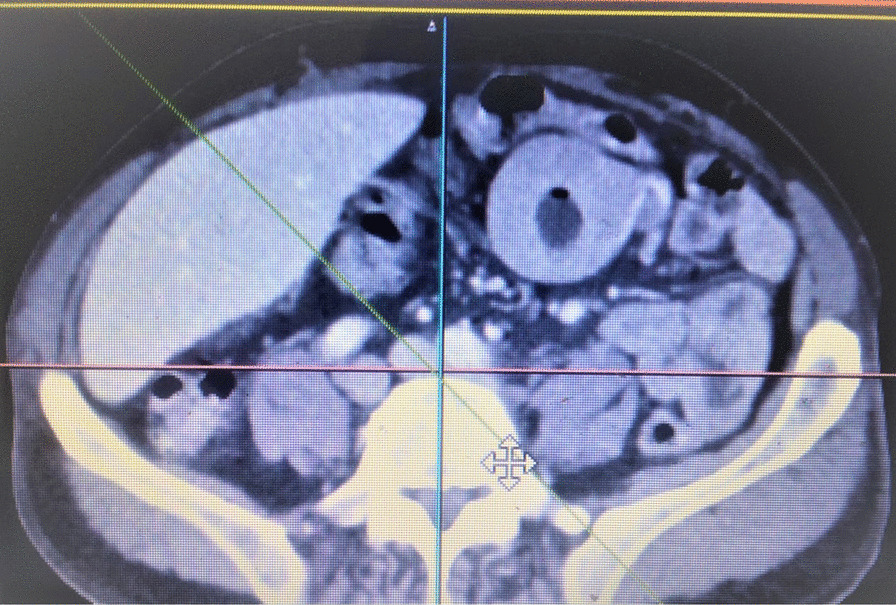
Computed tomography scan at the level of iliac bones showing circumferential mucosal thickening with heterogeneous enhancement suggestive of bowel metastasis

**Fig. 7:**
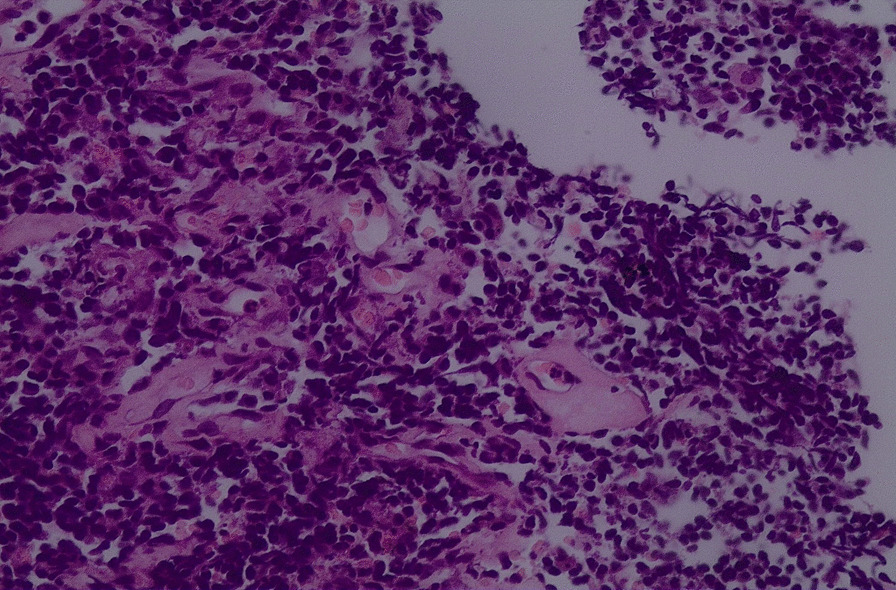
Histological section of the mass displaying a diffuse proliferation of small to intermediate sized cells with very scant cytoplasm and round to oval hyperchromatic nuclei

General examination revealed a wasted (BMI 17.6 kg/m^2^) man. He had a sinus bradycardia of 32 beats/minute and blood pressure of 117/66 mmHg on cardiovascular examination. Respiratory examination revealed features suggestive of right lung consolidation. Other systems were essentially normal. He underwent a number of hematological, biochemical, and serological tests, which revealed a normocytic normochromic anemia [hemoglobin (Hb) 11.1 g/dL] but otherwise normal. His echocardiography (ECHO) was essentially normal, however the electrocardiogram (ECG) showed features in keeping with the diagnosis of CHB, Fig. 1. Chest X-ray (CXR) showed a homogeneous opacification on the right side, and CT scan revealed a solid right lung mass with metastases to the heart, liver, bowels, and bone; Figs. 2, 3, 4, 5 and 6. Furthermore, he underwent bronchoscopy, which revealed an endobronchial mass occluding the bronchus intermedius. Histological examination of a section of lung biopsy taken during bronchoscopy confirmed the diagnosis of small cell carcinoma, Fig. 7. Correspondingly, a histological section of heart muscle tissue revealed similar findings to lung biopsy. The patient underwent dual chamber pacemaker implantation with successful sinus rhythm restoration. He made an informed refusal of chemotherapy and inevitably died in his home village 18 months post pacing. We received the death information from his son via a phone call, and an autopsy was not practical.

## Discussion

Despite its occurrence among patients with a wide tumor spread, cardiac metastasis is an uncommon phenomenon in clinical practice [1–3]. Tumors can spread to the heart by (i) direct extension, (ii) through the bloodstream, (iii) through the lymphatic system, and (iv) by intracavitary diffusion (that is through the inferior vena cava or the pulmonary veins) [3, 9]. The clinical presentation of cardiac metastases is either silent or vague, and largely depends on the infiltrated location and tumor burden [4, 10]. Seldom, cardiac involvement with neoplasm may manifest with dramatic life-threatening consequences including tamponade, myocardial infarction, stroke, and cardiogenic shock [1, 2, 4, 11]. Furthermore, malignant infiltration of the cardiac conduction system may result in numerous life-threatening arrhythmias including atrial fibrillation, ventricular tachycardia, and various degrees of atrioventricular blocks (including CHB) [12]. Notwithstanding the attributed morbidity and mortality to cardiac metastases, diagnosis is often reached during autopsy [1–11].

In spite of the improved diagnostic and therapeutic modalities, the incidence of cardiac metastases has increased in recent decades [1]. Specific physical or laboratory tests to detect cardiac metastases in a diffuse tumor state are lacking. A two-dimensional echocardiography (ECHO) may be useful in the detection of a cardiac involvement, however, the differentiation of a metastatic mass from a vegetation, thrombus, myxoma, or a primary cardiac tumor may be challenging [1]. This often necessitates the use of other imaging modalities such as computed tomography (CT) or magnetic resonance imaging (MRI) for better characterization of intracardiac masses. Additionally, a 3D or 4D doppler/duplex ultrasound would have been helpful in this scenario but owing to its high cost it was unavailable for use. Furthermore, electrocardiographic (ECG) findings, though generally nonspecific, may be pivotal in detecting arrhythmias and conduction defects [1].

To the best of our knowledge following an extensive literature search, this is the first ever case of complete heart block secondary to a small cell carcinoma to be documented in the literature. Other metastatic lung neoplasms that have been associated with CHB include anaplastic carcinoma, bronchogenic carcinoma, and squamous cell carcinoma [10, 13–15]. Despite several diagnostic limitations in a resource-limited setting such as ours, we are delighted to have reached the definitive diagnosis during the patient’s lifetime. Regrettably, like with many other malignant neoplasms, a diagnosis was reached in the extensive stage of the disease and arguably the prognosis would have remained somewhat similar even if this patient had agreed to chemotherapy.

## Conclusions

Despite its rarity, small cell carcinoma has the potential to metastasize to the heart, resulting in a complete heart block. Despite the remarkable advancements in medical diagnostics and management, lung cancers are often diagnosed in advanced stages with an inevitable grave prognosis. Timely pacing is crucial for restoration of a sinus rhythm and will somewhat improve the quality of life; however, the overall prognosis from the underlying tumor remains poor.

## Data Availability

Not applicable.

## Abbreviations

BMI: Body mass index
CHB: Complete heart block
CT: Computed tomography
CXR: Chest X-ray
ECG: Electrocardiogram
ECHO: Echocardiography
Hb: Hemoglobin
MRI: Magnetic resonance imaging
